# IP4M: an integrated platform for mass spectrometry-based metabolomics data mining

**DOI:** 10.1186/s12859-020-03786-x

**Published:** 2020-10-07

**Authors:** Dandan Liang, Quan Liu, Kejun Zhou, Wei Jia, Guoxiang Xie, Tianlu Chen

**Affiliations:** 1 Shanghai Key Laboratory of Diabetes Mellitus and Center for Translational Medicine, Shanghai Jiao Tong University Affiliated Sixth People’s Hospital, Shanghai, 200233 China; 2 Human Metabolomics Institute, Inc., Shenzhen, 518109 Guangdong China

**Keywords:** Metabolomics, Data analysis, Workflow, Software

## Abstract

**Background:**

Metabolomics data analyses rely on the use of bioinformatics tools. Many integrated multi-functional tools have been developed for untargeted metabolomics data processing and have been widely used. More alternative platforms are expected for both basic and advanced users.

**Results:**

Integrated mass spectrometry-based untargeted metabolomics data mining (IP4M) software was designed and developed. The IP4M, has 62 functions categorized into 8 modules, covering all the steps of metabolomics data mining, including raw data preprocessing (alignment, peak de-convolution, peak picking, and isotope filtering), peak annotation, peak table preprocessing, basic statistical description, classification and biomarker detection, correlation analysis, cluster and sub-cluster analysis, regression analysis, ROC analysis, pathway and enrichment analysis, and sample size and power analysis. Additionally, a KEGG-derived metabolic reaction database was embedded and a series of ratio variables (product/substrate) can be generated with enlarged information on enzyme activity. A new method, GRaMM, for correlation analysis between metabolome and microbiome data was also provided. IP4M provides both a number of parameters for customized and refined analysis (for expert users), as well as 4 simplified workflows with few key parameters (for beginners who are unfamiliar with computational metabolomics). The performance of IP4M was evaluated and compared with existing computational platforms using 2 data sets derived from standards mixture and 2 data sets derived from serum samples, from GC–MS and LC–MS respectively.

**Conclusion:**

IP4M is powerful, modularized, customizable and easy-to-use. It is a good choice for metabolomics data processing and analysis. Free versions for Windows, MAC OS, and Linux systems are provided.

## Background

Gas and liquid chromatography coupled with mass spectrometry (GC/LC-MS), among others, are the main technical approaches for metabolomics studies, as they are able to detect and quantify a large variety of metabolite molecules from cells, tissues and biological fluids [1]. However, it is challenging to get accurate and reproducible data processing results due to the complexity of mass spectra (MS) data. To extract information and knowledge from metabolomics data, several non-commercial computational tools have been successively generated and widely used in biological, agricultural and medical studies. For example, XCMS [2], MAIT [3], AMDIS [4], ADAP [5], and Metaboseek (https://metaboseek.com) [6], mainly focus on raw data preprocessing to generate peak intensity tables that can be further processed by other tools. MetabolAnalyze [7], metbaolomics [8], MetaboLyzer [9], and SIMCA-P [10], are designed for downstream statistical analysis using preprocessed peak intensity table. MSEA [11], ESEA [12], and Subpathway-GM [13], are specifically designed for pathway analysis with potential metabolic markers as inputs. To meet the evolving needs of the metabolomics research community, some integrated tools (with multiple interconnected functions), such as MetaboAnalyst [14], PiMP [15], Workflow4Metabolomics (W4M) [16], MZmine2 [17], MetaBox [18], XCMS online [19], MS-DIAL [20], and Galaxy-M [21], have been developed and have become increasingly popular in recent years. These tools are designed for comprehensive metabolomics data processing, allowing users to perform a nearly complete analysis by a single tool rather than several separate ones. However, there is still a quest for a more powerful, more comprehensive, and more friendly platform for both basic and advanced users.

Our research led to the development of a new platform, the Integrated Platform for Metabolomics data mining (IP4M). It covers all the core steps of metabolomics data mining, including peak picking, peak de-convolution, isotopes filtering, peak identification, data preprocessing (such as transformation, normalization, missing value imputation), basic statistical description, classification and biomarker detection (by unit- and multi-variant methods and machine learning methods), correlation analysis, cluster and sub-cluster analysis, regression analysis, receiver operating characteristic curve (ROC) analysis, pathway and enrichment analysis, and sample size and power analysis. Compared with existing multifunctional non-commercial tools, IP4M made advances in 3 aspects: (1) a reaction library (based on Kyoto Encyclopedia of Genes and Genomes (KEGG)) has been established and embedded. Based on this, some ratio variables will be generated and be included for differential and correlation analysis. These ratios, which partially reflect the bioactivity of metabolic enzymes and reactions, may provide more information than that of traditional metabolomics data. (2) A new method, Generalized coRrelation analysis for Metabolome and Microbiome (GRaMM) [22], which was designed and developed recently for the inter-correlation detection of metabolome and microbiome data, has been embedded. (3) The pathway analysis module with richer knowledgebase and extended algorithms is beneficial to data interpretation. Pre-set workflows for a quick and reproducible analysis that does not require complex parameter settings or computer programming are included. However, IPM4 also offers independent modules and sufficient parameters for advanced users to obtain a customized and more refined analysis.

Four data sets, 2 generated from a GC–MS platform and 2 from a LC–MS platform, were used to evaluate the performances of IP4M. For each platform, 1 data set derived from a mixture of known standards and 1 derived from serum samples were employed. The standard mixture data sets were used to test the performances of peak picking and annotation and the real world data sets were used to test the performances of differential analysis, correlation analysis, and pathway analysis. Several widely used tools, including MetaboAnalyst, MetaboAnalystR, W4M, Galaxy-M, XCMS online, MZmine2, MS-DIAL, metabox and Metaboseek, were involved in the comparisons. Currently, IP4M has been used successfully for more than 100 data sets from 20+ labs.

## Methods

IP4M was jointly developed using the languages of Java, Perl, and R. The Graphical User Interface (GUI) was developed with Eclipse rich client platform (RCP) for the development of rich client desktop applications. The source code and demo data sets are provided at https://github.com/IP4M. The software (Windows/Mac/Linux versions) and manuals can be downloaded via https://ip4m.cn. When the sample size was over 1000 and each raw data file was over 100 MB size, it would take a long time (over 6 h) for both LC–MS data and GC–MS data preprocessing. Minimum computer hardware configuration with CPU over 3.0 GHz, 2 cores, and RAM over 8 GB is recommended.

**Fig. 1:**
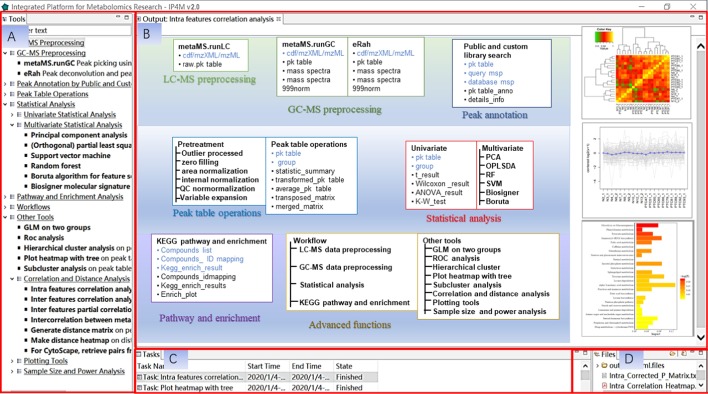
Interface and function modules of IP4M. **a** The tools window contains a directory tree for function navigation. **b** The main window to display parameters and results. **c** The tasks window to list tasks and their status. **d** The files window to list output files. The major functions and inputs/outputs (in blue/black) of IP4M are shown in (**b**)

IP4M contains 62 independent functions. As shown in Fig. 1, they are categorized into 8 modules, including LC–MS data preprocessing, GC–MS data preprocessing, peak annotation, peak table operations, statistical analysis, pathway and enrichment analysis, workflows, and other tools.

### Inputs and outputs

Raw data from both GC–MS and LC–MS instruments, in mzML, mzXML and/or netCDF formats, are supported. Other files (e.g., peak table, sample information, compound list) in tab-delimited text format are supported, with variables in columns and samples in rows. The msConvert tool of free ProteoWizard is recommended for format conversion. All the results are exported as.txt (data and tables) or.pdf (figures) files. More details and examples of output results are shown in the user manual and the help pages of GUI.

### LC–MS and GC–MS data preprocessing

Two peak picking methods are introduced in IP4M for raw data preprocessing, metaMS and eRah. metaMS [23], which is based on the latest version (V3.8.1) of the XCMS package and CAMERA package (V1.42.0), can be used to preprocess both LC- and GC-MS data sets. It makes a technical advance by providing a series of optimized parameter settings for diverse experimental designs and instruments. The CAMERA package of metaMS is useful for isotope and pseudospectra identification as well as, artefacts removal. Comparatively, the eRah [24] can only preprocess GC–MS data sets and it usually provides more peaks at the cost of longer running time and higher CPU supports, compared to metaMS (see comparisons in Table 2). The peak picking step, which is based on blind source separation (BSS) and multivariable chromatography deconvolution, is probably the most time consuming step of eRah. metaMS does not contain the deconvolution step for peak picking.

### Peak annotation

For peak annotation, both public libraries and in-house databases are supported by IP4M. Many public databases, including Human Metabolome Database (HMDB), Golm Metabolome Database (GMD), and National Institute of Standards and Technology (NIST), have been incorporated into IPM4, with ~ 15,000 compounds in total. The exact molecular mass (for LC–MS) and spectral similarity (for GC–MS) are matched with those in public and in-house libraries. Retention time (RT) serves as an optional criterion if it is provided in an in-house library. The best hit with RT error (in second), M/Z error (in ppm or Dalton), and/or possible structures (fragments and adducts for LC–MS; mass spectrum list for GC–MS) will be reported. Several top candidates with supporting information will also be reported in the extended file for advanced users.

### Peak table operations

This module contains many simple but useful functions for outlier detection, missing value imputation, data structure pretreatment and variable extension. Outliers (data points exceeding ± 3S.D. of the corresponding variable) are replaced by the maximum value of the remaining data. Missing values can be imputed or replaced by the minimum strategy, the KNN method, or the ‘qrilc’ algorithm [25, 26]. Three frequently used normalization approaches (by total signal intensity of each sample, by intensity of internal standard, and by quality control samples [27]) have been implemented. IP4M also provides log-transformation and z-score transformation to linearize data structure. Other small functions such as matrix transpose, retrieving target rows, samples or variables combination and basic statistics calculations are also integrated, with the goal of facilitating data integration. Notably, a “variable expansion” function that allows one to generate a series of ratio variables is incorporated into this module. The ratio of a product to a substrate of a specific metabolic reaction is usually taken as a marker of the bioactivity of the metabolic reaction and/or the catalytic enzyme of this reaction. A local metabolic reaction pair database was established by extracting corresponding information from KEGG and the ratios of products and substrates within the embedded reaction pair database will be generated. These ratio variables with enhanced information derived from the original metabolomics data set can be involved in subsequent analysis. All of the above functions contribute to a high quality peak table for downstream analysis.

### Statistical analysis

Common univariate tests, parametric or nonparametric tests, for two groups or more than two groups, are introduced for potential biomarkers identification. Three multiple testing correction methods, Bonferroni, Holm and the false-discovery rate (FDR), are offered. FDR is the default one as it is more suitable for most metabolomics studies where more than one metabolite is expected to be screened out [1].

In addition to univariate analysis, which does not consider the effect of collinearity among variables, IP4M offers several multivariate analysis methods, including the conventional linear ones (principal component analysis (PCA) and (orthogonal) partial-least squares discriminant analysis ((O)PLS-DA)), some popular machine learning methods (Random Forest (RF), support vector machines (SVM)) and some integrated methods (biosigner [28] and Boruta [29]) for "potential biomarkers" screening and re-screening. For PCA, PLS-DA and OPLS-DA, their scores, loadings, and permutation plots, the coordinate values of these plots, and the OPLS-DA VIP values of the variables will be exported. RF is a nonlinear algorithm and can handle small differences and large noise without or with little over-fitting. The ranked importance of variables for group separation is calculated by an ensemble of classification trees and the majority vote of the ensemble. The SVM algorithm aims to find a nonlinear decision curve to separate samples using a maximum margin hyperplane. It constructs the final decision function by a small number of support vectors and kernel functions. Four kernel functions, linear, polynominal, radial and sigmoid kernel, are available in IP4M. The biosigner is supposed to screen significant variables based on the results of the 3 aforementioned methods (PLS-DA, RF, SVM) collaboratively. Boruta is supposed to re-screen potential biomarkers from significant variables provided by other methods. The variables with significant distinguishing ability than that of permutated ones will be confirmed as potential biomarkers. The results of biosigner and Boruta are likely more robust as the results of multiple methods are jointly considered.

Every single method, as a separate function, can be conducted independently. In the “statistical analysis” workflow, all the methods are conducted one by one on the same inputs automatically. PCA and (O)PLS-DA, SVM, RF, Boruta, and biosigner are based on the R package “ropls” [30], “e1071” [31, 32], “randomForest” [33], “Boruta” [33–35], and “biosigner” [36] respectively.

### Pathway and enrichment analysis

This module is based on the corresponding module of MetaboAnalyst 4.0 [14, 37], the pioneering and leading tool of pathway and enrichment analysis. It is used to identify the impact of metabolites and the pathways they are involved in and to evaluate their associations with disease/SNP/drug metabolism and many other functional and biological contexts. Compared with MetaboAnalyst, IP4M has two improvements. First, we have greatly expanded the species and pathway libraries A total of 5871 pathways covering 67 species (list in Addional file 1: Table S11, there are 1600 pathways of 25 species in the latest MetaboAnalyst), such as Chimpanzee, Nomascus leucogenys (northern white-cheeked gibbon), Macaca fascicularis (crab-eating macaque), Cricetulus griseus (Chinese hamster), Bubalus bubalis (water buffalo), and Ovis aries (sheep), are loaded as a knowledgebase for this module. All the information for these libraries was extracted from KEGG. Each species has its own pathway information, including information on reaction equations, substrates, products, key enzymes, and reversible and irreversible reactions and so on. Second, IP4M not only provides the existing "out-degree centrality" and "relative—betweenness centrality" algorithms (provided by MetaboAnalyst) for pathway topology analysis, but also offers 5 other algorithms, including "total-degree centrality", "out/in/total closeness centrality", and "eigenvector centrality". Specifically, the degree centrality [38] of the node is the number of nodes connected to it. When the connection (a metabolic reaction) has a direction, it will be considered in the node centricity calculation. The “in-degree” is a count of the number of connections directed to the node and the “out-degree” is the number of connections that the node directs to others. The “total-degree” is the sum of in- and out-degrees, regardless of direction. Betweenness Centrality [39] is the number of shortest paths through a node. Closeness centrality [40] is the reciprocal of the sum of the distances from a node to all the other ones connected to it. It measures how close a node is to other nodes. Unlike all the other algorithms, “Eigenvector Centrality” [41] takes a more systematic approach to measuring a node's impact on a network which considers the centrality of the node itself and all of its neighbors. For two nodes with the same number of connections, the one connected to more important nodes will achieve a higher Eigenvector Centrality. To sum up, the focuses of different pathway topology algorithms are different. Joint usage of multiple algorithms is recommended for complicated cases to ensure a reliable result. The default algorithm in IP4M is the “Eigenvector Centrality”.

### Workflows

Typical metabolomics data mining pipelines are packaged into four workflows for a quick and reproducible analysis: LC–MS and GC–MS peak picking and annotation (from raw data to peak table), statistical analysis (from peak table to statistical analysis results), and pathway and enrichment analysis (from compound names to pathway and enrichment results). Numerous parameters have been hidden and default values are optimized for core parameters. The output results are also slightly simplified. This module is specially designed for basic users and batch applications.

### Other functions

Apart from basic computational metabolomics functions, IP4M also provides many popular functions that may be conductive to metabolomics studies, including inter/intra-correlation analysis, clustering (hierarchical clustering and sub-clustering) analysis, distance analysis, linear regression analysis, ROC analysis, power and sample size analysis, and some plotting tools. In addition to common correlation methods (Pearson, Spearman, Kendall, and partial Spearman), a newly published correlation algorithm, GRaMM [22], is provided. This algorithm is designed specifically for the inter-correlation detection between metabolome and microbiome data. It is able to identify both linear and nonlinear correlation pairs with the consideration of known confounders. The input files (.txt) include a metabolite matrix (e.g., a peak table), a microbial abundance matrix (e.g., a 16 s rRNA taxa abundance data or a metagenome function data) and a confounding variable matrix (optional). The outputs include the correlation type (linear or nonlinear), the correlation strength (the r value), and the correlation significance (the *p* and corrected *p* values) for each metabolite–microbe pair. The sub-cluster analysis groups the inputted peaks into several clusters and shows the variance of each cluster across samples/groups. Seven commonly used distance metric approaches (Euclidean, correlation, Minkowski, Canberra, binary, Manhattan, maximum distance) and seven clustering methods (ward, single, complete, average, mcquitty, median, centroid clustering) are available. IP4M can also retrieve pairs from a correlation matrix (an output of correlation analysis) according to a specific criterion and the resulting files can be imported directly into Cytoscape for network construction. The multiple “power and sample size analysis” functions are consistent with conventional power analysis which provides a reference for experimental design and experimental results evaluation, depending on the effect size, significance level, power of test, and type of test. The Venn diagram analysis (up to 6 groups) and many graphic tools such as heatmap, pairwise scatter, box, line, and bar chart are provided for result visualization.

### Function comparisons with existing tools

The functions of IP4M and 7 widely used integrated platforms, such as MetaboAnalyst (v4.0), W4M (v3.3), Galaxy-M, XCMS online (v3.7.1), MZmine2 (v2.5.1), Metabox (v1.2) and MS-DIAL (v4.24), are compared in Table 1. For peak picking, no one tool can work with all types of data sets. For LC–MS preprocessing, all the tools, except for MZmine2 and MS-DIAL, are based on the XCMS package (on different versions). For the 6 tools with the GC–MS preprocessing function, IP4M and W4M are based on the same R packages. MetaboAnalyst relies on a third-party platform which has an inputs/outputs style that is different from MetaboAnalyst and can only process a limited number (< 30) of samples. The workflow and methods of XCMS online for GCMS data set processing appear to be the same as that of LC–MS since the settable parameters are the same and no additional parameters for de-convolution exist. MZmine2 provides numerous parameters and professional visualizations as it is designed for advanced users who are familiar with, or at least have general understanding of, the measurement theory and data characteristics of MS. MS-DIAL can handle multiple types of raw data, and data from multiple vendors (via the AbfConverter for format conversion). It can perform deconvolution using the MS2Dec algorithm and provides various libraries for peak identification (including lipids). Furthermore MS1, XCMS online, MZmine2 and MS-DIAL are also compatible with MSMS data sets and Galaxy-M is the only tool capable of preprocessing direct infusion mass spectrometry (DIMS) data sets. For peak table pretreatment, IP4M is superior to the others with the most functions and all the other tools are comparable. For statistical analysis, pathway analysis, and other functions, IP4M and MetaboAnalyst are comparable and are more powerful than the others. IP4M is the only tool with the function “ratio variable generation”, “Boruta” and “GRaMM”. MetaboAnalyst has a stronger support to multi-omics integration analysis and supports time series analysis. Taken together, MS-DIAL, MZmine2, IP4M, and W4M are slightly better for MS data preprocessing, whereas MetaboAnalyst, IP4M, and W4M provide more comprehensive functions for subsequent analysis. IP4M is comparable to or better than existing tools, while it is limited in cross-omics integrated analysis.

**Table 1:** Function comparisons of IP4M and other 6 tools

Function tool	IP4M	MetaboAnalyst	W4M	Galaxy-M	XCMS online	MZmine2	MetaBox	MS-DIAL
Raw data preprocessing								
LC–MS	√	√	√	√	√	√	–	√
GC–MS	√	√	√	–	√	√	–	√
NMR	–	√	√	√	–	–	–	–
MS/MS	–	–	–	–	√	√	–	√
DIMS	–	–	–	√	–	–	–	–
Peak annotation	√	√	√	√	√	√	–	√
Peak table operations								
Normalization	√	√	√	√	√	√	√	√
Scaling	√	√	√	√	√	√	√	√
Zero filling	√	√	√	√	√	√	√	√
Transformation	√	√	√	√	–	–	√	√
Ratio variable generation	√	–	–	–	–	–	–	–
Basic statistical summary	√	–	√	–	–	–	–	√
Retrieve rows	√	–	–	–	–	–	–	–
Samples and variables merging	√	–	√	–	–	√	–	–
Statistical analysis								
Univariate analysis	√	√	√	√	√	√	√	√
Multivariate analysis								
PCA/(O)PLS-DA	√	√	√	√	√	√	√	√
SVM	√	√	–	–	–	–	–	–
RF	√	√	–	–	–	–	–	–
Biosigner	√	–	√	–	–	–	–	–
Boruta	√	–	–	–	–	–	–	–
Pathway and enrichment analysis	√	√	–	–	√		√	√
Integrated workflows	√	–	√	√	–	√	–	–
Other functions								
Correlation/distance	√	√	√	–	–	–	√	√
Regression	√	–	–	–	–	√	–	–
ROC analysis	√	√	–	–	–	–	–	–
Hierarchical cluster	√	√	√	–	√	√	–	√
Plotting tools	√	√	√	–	√	√	–	–
Power/sample size	√	√	√	–	–	–	√	–
Network analysis	–	√	–	–	–	–	–	–
Omics data integration analysis	√	√	–	–	√	–	√	–
Time series analysis	–	√	–	–	–	–	–	–
Developing language	R, Java, Perl	R, Java	R	R, Python, Matlab	R	Java R	R	C#,
Local GUI/web server	Local GUI; Windows/Linux/OS	Web server; Local R package	Web server	Web server	Web server	Local GUI; Windows/Linux/OS	Local GUI; Windows/Unix/Linux	Local GUI; Windows

“√” means yes and “–” means no or N/A

IP4M is a local GUI-based tool considering the heavy burden and risk of big data uploading, saving, and privacy. To make it easier to use, we tried our best to pack in as many supporting packages as possible. For the Windows version, neither installation nor extra configuration is required for running. For the Mac and Linux versions, a one-step environment setting is required (see manuals).

## Results and discussion

Four data sets, 2 obtained from a GC-MS platform and 2 from a LC-MS platform were used to illustrate the performance of IP4M. Among them, 2 were mixture standard data sets that were supposed to evaluate the performances of peak picking and peak annotation. The other two were real world data sets (do you mean serum samples here) from an animal experiment which were used to demonstrate the performances of peak table statistical analysis and interpretation. The details of these data sets are available in the supplementary information (SI).

### Comparisons based on standard mixture data sets

A published LC–MS standard mixture data set [42] with 152 standards and a GC–MS standard mixture data set (SI) from our lab with 33 standards were used to test the peak picking and annotation performances of several tools. All the standards were able to be found by at least one of the tools.

The LC–MS data set was processed separately by MetaboAnalystR (v2.0), W4M (v3.3), Galaxy-M, XCMS online (v3.7.1), MZmine2 (v2.5.1), MetaboSeek (0.9.5), MS-DIAL (v4.24) and IP4M (v2.0), using the same computer. Considering the difference in settable parameters of the tools, 4 key parameters were kept consistent: SNR = 3, mzdiff = 0.05, bw = 10, and corr_eic_th = 0. Other parameters were set as default values of the tools (see SI for detailed settings of each tool). Table 2 lists the total features detected and the number of true features provided by different tools. Features were matched with the same reference library (Addional file 1: Table S1) based on the difference of m/z and retention time tolerance (ppm < 10 or m/z toleration < 0.005 Da and retention time tolerance < 0.3). As Table 2 shows, the numbers of matched true features for all the tools, except Metaboseek (n = 112), were comparable (n = 68–89). The performance of IP4M is moderate (n = 76). Although the core package for LC–MS dataset processing of all the tools (excepting for MZmine2) was XCMS, the results from different tools were different, probably due to their different default parameters. In IP4M, all the key parameters of XCMS were adjustable and advanced users can refine their results by tuning the parameters carefully. The default parameters suitable for most cases are also provided for basic users.

**Table 2:** Number of features picked and matched with standard mixture data sets using different tools

Tool	Number of total features	Number of true features	Time spent (h)
LC–MS			
IP4M (metaMS)	5150	76	0.5
W4M	4102	68	0.5
Galaxy-M	6021	74	0.5
XCMSonline	6339	79	0.6
MZmine2	3033	81	1.5
MetaboanalystR	5386	84	2.0
Metaboseek	3863	112	0.5
MS-DIAL	6765	89	4.0
GC–MS			
IP4M/W4M (metaMS)	84	27	1.3
IP4M (eRah)	188	33	1.0
MS-DAIL	241	25	0.5
MZmine2 (ADAP)	130	28	2.0

The computer used: Windows 10, CPU = 3.4 GHz, 4 cores, and RAM = 16 GB

The GC–MS data set was separately processed by IP4M, MZmine2 and MS-DIAL with default parameters, and the results are shown as Table 2. The same in-house standard database (Addional file 1: Table S2, the database with 33 standard mass spectrum information) was used for mass spectral matching and metabolite annotation (similarity of mass spectrum > 0.7, retention time tolerance < 0.3). As Table 2 shows, eRah (IP4M) out-performed metaMS (W4M, IP4M), ADAP (MZmine2) and MS-DIAL, and all 33 standard metabolites were identified within the shortest time. The results of W4M were not shown separately as the corresponding packages of W4M and IP4M are the same and so were the results. The results of XCMS online were not shown as well, as its embedded library (METLIN) is different to the others and only 9 metabolites were identified correctly.

### An application using real world data sets

**Fig. 2:**
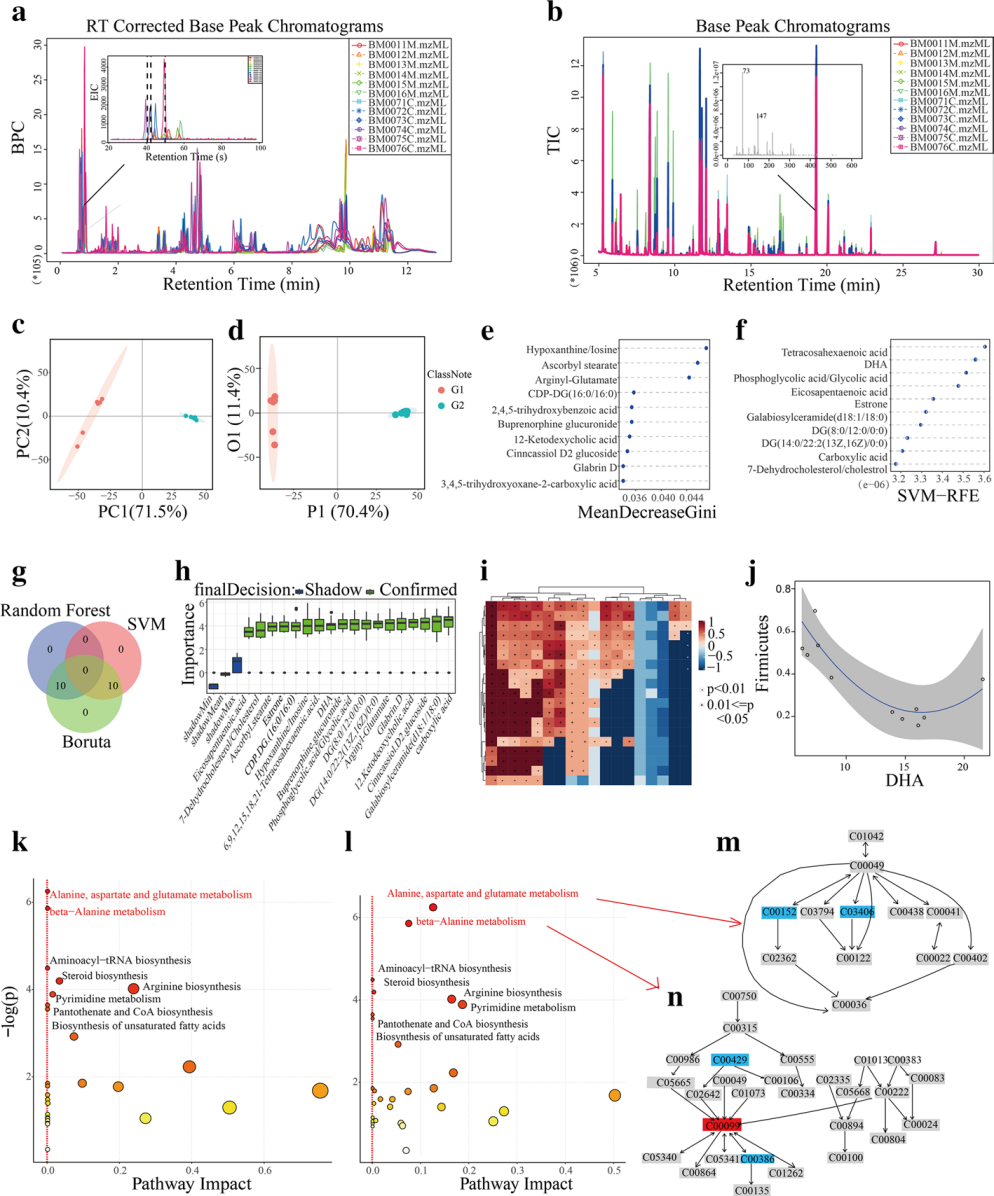
Some results of IP4M using a real world data set. ‘G1’ indicates the group of 1-week-old mice and ‘G2’ indicates the group of 7-week-old mice. **a**, **b** BPCs of LC–MS and TICs of GC–MS data sets derived from murine brain metabolic profiles. **c**–**h** Some results of multivariate differential analysis: PCA scores plot (**c**); OPLS-DA scores plot (**d**); the top 10 potential markers provided by RF (**e**) and SVM (**f**); the venn plot (**g**) of potential markers from RF, SVM and Boruta; and the validation boxplot (**h**) of Boruta based on the union set of the top 10 markers from RF and SVM. **i**, **j** Results of the correlation analysis: r value heatmaps GRaMM (**i**); scatter plot with nonlinear fitting curve of the correlated pair “DHA ~ *Firmicutes*” derived from GRaMM (**j**). **k**–**n** Some results of pathway analysis: the bubble plot of pathway analysis using “relative—betweenness centrality” algorithm (**k**) and “eigenvector centrality” algorithm (**l**). Each bubble corresponds to a metabolic pathway. The x-coordinate indicates the extent of pathway influence (Pathway Impact). The point size is related to Pathway Impact of the pathway. The ordinate represents the negative logarithm of the *p* value obtained from the enrichment analysis. Pathways with *p* < 0.05 are labeled. **m**, **n** The network diagrams of two significant pathways. Up and downregulated metabolites (G2 versus G1) are highlighted in red and blue respectively

Two real world data sets from our previous animal study were used to validate the performance of the peak table statistical analysis and interpretation [43]. The study was approved by the ethical committee of our hospital. The brain metabolic profiles of 12 normal Wistar rats (2 groups, 6 one-week-old and 6 seven-week-old) were acquired using both UPLC/QTOF-MS (Waters, U.S.A.; positive and negative modes) and GC/TOF–MS (Leco, U.S.A.) platforms. Please refer to the original paper for detailed information of the animal experiment and data acquisition. The LC/MS and GC/MS data sets were imported into IP4M for peak processing. In total, 8967 (the total ion chromatogram is shown in Fig. 2a) and 189 peaks (the basic peak chromatogram is shown in Fig. 2b) were detected using both the metaMS runLC method and metaMS runGC methods, respectively. Among them, 721 and 118 were identified using HMDB and NSEN (a self-integrated library based on NIST, EPA, and NIH library) respectively. These identified metabolites were combined and extra 65 ratio variables were generated and added into subsequent statistical analysis. Student’s t test and several multivariate statistical analysis methods were conducted on all the variables. The total ion chromatograms (TICs), basic peak chromatograms (BPCs), and extracted ion chromatograms (EICs) of some of the raw data sets are shown as Fig. 2a, b. The PCA and OPLS-DA score plots derived from all the variables are shown as Fig. 2c, d which are similar to that of other tools (SI). There are clear group separations in both plots. Overall, 422 variables were identified as differential ones between the two groups, with the unpaired student’s *t* test *p* < 0.05 and the OPLS-DA VIP > 1. To narrow down the potential biomarkers, we analyzed these differential variables further using RF and SVM. The top ten important variables from RF and SVM are listed as Fig. 2e, f respectively. These 20 important variables, including 3 ratio variables, were imported into Boruta for validation and all of them were confirmed as potential biomarkers (Fig. 2h). Compared with the 7-week-old rats, the younger group showed higher levels of fatty acids, phospholipids, ratio of hypoxanthine to inosine and ratio of 7-dehydrocholesterol to cholesterol, which play important roles in growth. Among these metabolites, DHA [44, 45], a highly unsaturated fatty acid, is essential for brain nutrition, as it can promote foetal brain cell development and neuroretinal development. 7-dehydrocholesterol [46–48] converted from cholesterol can then be converted into vitamin D3 under ultraviolet light, which has the biological activity of regulating calcium and phosphorus metabolism.

Furthermore, we analyzed the inter-correlations between the 20 potential biomarkers and 18 bacterial phyla (derived from the intestinal contents of the same samples) using the newly developed GRaMM. Age (the week age of rats) was taken as the confounding variable. The correlation coefficients and *p* values are shown in heatmaps (Fig. 2i). The correlation pairs identified by GRaMM are in line with some previous reports [22, 43]. For example, the metabolite DHA and microbial phylum *Firmicutes* showed a significant nonlinear relationship. This kind of nonlinear pair is rarely detected by common methods (e.g., Pearson, Spearman, linear regression). It is well known that many microbes under Firmicutes are related to digestive tract diseases [49, 50]. For example, *Ruminococcus gnavus* has the function of pro-oxidation and C3, 7, 12 hydroxyl isomeric [51]. It can produce iso-bile acids, and iso-bile acids can reduce DCA toxicity [52]. More studies, especially in vivo and in vitro experiments are needed to validate the relationship of this pair.

Finally, pathway and enrichment analysis were carried out on the union set of top 100 most important variables ranked by SVM and RF. The results of two typical topological analysis algorithms are shown in Fig. 2k (using “relative-betweeness centrality”) and Fig. 2l (using “eigenvector centrality”). They found the same eight differential pathways (*p* < 0.05). This means that these 8 pathways and the metabolites within them were significantly altered between the young and adult rats. However, the impact values of these pathways (the x-axis), which were affected by the importance of differential metabolites in corresponding pathways, were different. This is because the importance of metabolite in the network calculated by betweenness centrality (Fig. 2k) is based on the number of shortest routes through this metabolite (node), while eigenvector centrality (Fig. 2l) is based on the importance of itself and all of its surrounding nodes. The KEGG pathway figures of alanine, aspartate and glutamate metabolism and beta–alanine metabolism are illustrated as Fig. 2m, n. Beta–alanine metabolism is mainly in the brain and muscles, and the final product of normal metabolism is acetic acid [53]. As a neurotransmitter or hormone regulator, it can regulate metabolism in the body [54] and improve the body's ability to exercise [55], memory [56].

## Conclusion

IP4M is an integrated platform for MS based untargeted metabolomics data mining. It is open source and easy to use. The strength of IP4M is that it has comprehensive functions and useful tools, a rich knowledgebase, and options for customizable operations and integrated workflows. However, currently, IP4M is a stand-along local tool. We are planning to construct a server cluster-based online platform (iIP4M) to serve more users and run bigger samples size. In addition to MS1 data, MSMS data preprocessing and targeted metabolite quantification will also be added to the data preprocessing module. The interactive operation and visualization for peak checking and modification of this module will be further improved. Current functions for multi-omics data integrative analysis are limited. More functions are need to be incorporated along with the rapid development of this field. In conclusion, IP4M, a comprehensive, user-friendly, and open source platform, may serve as an attractive alternative tool for metabolomics data mining.

## Availability and requirements

- *Project name* IP4M.
- *Project home page* https://IP4M.cn.
- *Operating system(s)* Windows 2007 or 2010; Ubuntu 16.04 or 18.04; macOS Catalina 10.15.
- *Hardware* CPU > 3.0 GHz; Memory > 8 Gb.
- *Programming language* Java, Perl, R, Eclipse RCP.
  *Programming language* Java, Perl, R, Eclipse RCP.
- *Other requirements* For Windows users: no installation is required. Please download the.zip file and click the only.exe file to launch IP4M directly. R, Perl, and python environments are not required. Administrator privileges are required. For Linux and Mac users: a few steps for environment configuration are required.
- *License* GNU GPL.V3.
- *Any restrictions to use by non-academics* None.

## Supplementary information

**Additional file 1**. The supplementary information includes 4 test data sets, the parameters of  comparison softwares, the new species libraries added into IP4M for enrichment analysis, and the typical multivariable analysis reselts of other softwares.

## Data Availability

Source code, data sets and supplementary materials are available on GitHub (https://github.com/IP4M).

## Abbreviations

MS: Mass spectra
ROC: Receiver operating characteristic curve
GUI: Graphical user interface
RCP: Rich client platform
LC-MS: Liquid chromatograph-mass spectrometer
GC-MS: Gas chromatograph-mass spectrometer
NMR: Nuclear magnetic resonance
RT: Retention time
KEGG: Kyoto Encyclopedia of Genes and Genomes
HMDB: Human metabolome database
Golm: Metabolome database
NIST: National institute of standards and technology
M/Z: Mass-to-charge ratio
KNN: K-nearest neighbor
FDR: The false-discovery rate
PCA: Principal component analysis
(O)PLS-DA: (Orthogonal) partial-least squares discriminant analysis
RF: Random forest
SVM: Support vector machine
VIP: Variable importance for the projection
SNP: Single nucleotide polymorphism
csv: Comma-separated values
GC-TOF/MS: Gas chromatography-time-of-flight mass spectrometry
DIMS: Direct infusion mass spectrometry
NSEN: A self-integrated library based on NIST, EPA, and NIH library
TIC: Total ion chromatograms
BPC: Basic peak chromatograms
EIC: Extracted ion chromatograms
SNR: Signal/Noise ratio
ppm: Parts per million
mzdiff: Difference in m/z (mass-to-charge)
bw: Bandwidth
