# Supplementary material for: IP4M: an integrated platform for mass spectrometry-based metabolomics data mining
Source: BMC Bioinformatics. 2020 Oct 7;21:444. doi: 10.1186/s12859-020-03786-x (PMC7542974; doi:10.1186/s12859-020-03786-x)
Supplement: Supplementary file 1 — Additional file 1. The supplementary information includes 4 test data sets, the parameters of comparison softwares, the new species libraries added into IP4M for enrichment analysis, and the typical multivariable analysis reselts of other softwares. [file 12859_2020_3786_MOESM1_ESM.docx]

IP4M: An integrated platform for mass spectrometry-based metabolomics data mining

Dandan Liang^1^, Quan Liu^2^, Kejun Zhou^2^, Wei Jia^1*^, Guoxiang Xie^2*^, and Tianlu Chen^1*^

**Contact:** [chentianlu@sjtu.edu.cn](mailto:chentianlu@sjtu.edu.cn); [xieguoxiang@hmibiotech.com](mailto:xieguoxiang@hmibiotech.com); [wjia@sjtu.edu.cn](mailto:wjia@sjtu.edu.cn)

^1^Shanghai Key Laboratory of Diabetes Mellitus and Center for Translational Medicine, Shanghai Jiao Tong University Affiliated Sixth People’s Hospital, Shanghai 200233, China

^2^Human Metabolomics Institute Inc., Shenzhen, Guangdong 518109, China

Contents

[Materials 3](#_Toc45487462)

[Test datasets 3](#_Toc45487463)

[1. LC-MS standard mixture dataset 3](#_Toc45487464)

[2. GC-MS standard mixture dataset 6](#_Toc45487465)

[3. LC-MS and GC-MS real-world datasets 7](#_Toc45487466)

[Results 7](#_Toc45487467)

[1. Parameters of other tools 8](#_Toc45487468)

[2. Pathway and enrichment analysis 14](#_Toc45487469)

[3. Typical multivariable analysis results of some other tools 18](#_Toc45487470)

[**Reference** 18](#_Toc45487471)

Materials

Test datasets

1. LC-MS standard mixture dataset

We used a published standard mixture data set[1] derived from a SCIEX TripleTOF 6600 to test peak picking performance of different tools. There are a total of 152 known metabolites (in the retention time range of 15 ~ 25 minutes) in the samples.

Table S1. Benchmark feature list and quantification table for the TripleTOF 6600 dataset

| Feature number | Compound formula | Adduct | m/z | RT (min) | Fold change | p-value | Concentration ratio |
| --- | --- | --- | --- | --- | --- | --- | --- |
| 1 | C24H30F2O6 | +H | 453.2079 | 15.8 | 0.05 | 0.000 | 1/16 |
| 2 | C18H14Cl4N2O | +H | 416.9905 | 24.58 | 3.91 | 0.000 | 4/1 |
| 3 | C21H26N2S2 | +H | 371.1612 | 18.84 | 0.55 | 0.000 | 1/2 |
| 4 | C21H24F3N3S | +H | 408.1716 | 17 | 16.61 | 0.000 | 16/1 |
| 5 | C22H26F3N3OS | +H | 438.182 | 15.81 | 0.28 | 0.000 | 1/4 |
| 6 | C18H15Cl3N2S | +H | 397.0093 | 22.58 | 15.95 | 0.000 | 16/1 |
| 7 | C18H15Cl3N2O | +H | 381.0322 | 21.68 | 19.29 | 0.000 | 16/1 |
| 8 | C14H21N3O3S | +H | 312.1378 | 16.24 | 5.18 | 0.000 | 4/1 |
| 9 | C23H31NO2 | +H | 354.2429 | 20.1 | 0.28 | 0.000 | 1/4 |
| 10 | C25H32ClN5O2 | +H | 470.2307 | 16.48 | 0.41 | 0.025 | 1/2 |
| 11 | C21H35NO | +H | 318.2793 | 21.44 | 1.84 | 0.004 | 2/1 |
| 12 | C25H31NO6 | +H | 442.2221 | 17.92 | 18.53 | 0.000 | 16/1 |
| 13 | C25H37NO4 | +H | 416.2774 | 18.99 | 1.16 | 0.155 | 1/1 |
| 14 | C10H8F2N4O | +H | 239.0743 | 15.17 | 1.36 | 0.006 | 1/1 |
| 15 | C19H30O3 | +H | 307.2267 | 17.53 | 1.19 | 0.015 | 1/1 |
| 16 | C15H15NO3 | +H | 258.1127 | 16.84 | 1.64 | 0.059 | 1/1 |
| 17 | C15H13NO3 | +H | 256.0969 | 15.53 | 1.17 | 0.048 | 1/1 |
| 18 | C12H15N5O3 | +H | 278.1214 | 24.24 | 1.37 | 0.003 | 1/1 |
| 19 | C27H31N7OS | +H | 502.2384 | 23.18 | 1.40 | 0.008 | 1/1 |
| 20 | C27H37N3O7S | +H | 548.2423 | 22.09 | 1.34 | 0.015 | 1/1 |
| 21 | C21H26O2 | +H | 311.2005 | 21.85 | 1.08 | 0.379 | 1/1 |
| 22 | C14H18N2O | +H | 231.1493 | 24.21 | 1.11 | 0.027 | 1/1 |
| 23 | C24H30O6 | +H | 415.2118 | 22.78 | 1.11 | 0.060 | 1/1 |
| 24 | C25H24N6O2 | +H | 441.2036 | 21.57 | 1.14 | 0.199 | 1/1 |
| 25 | C23H22F7N4O6P | +H | 615.1235 | 24.23 | 1.26 | 0.003 | 1/1 |
| 26 | C19H18N2O4S | +H | 371.1062 | 24.73 | 1.33 | 0.007 | 1/1 |
| 27 | C40H57N5O7 | +H | 720.4335 | 24.74 | 0.94 | 0.701 | 1/1 |
| 28 | C46H62N4O11 | +H | 847.4493 | 22.47 | 0.86 | 0.045 | 1/1 |
| 29 | C15H9FN2O3 | +H | 285.0669 | 24.8 | 1.13 | 0.172 | 1/1 |
| 30 | C19H33NO2 | +H | 308.2586 | 24.7 | 1.14 | 0.027 | 1/1 |
| 31 | C22H24FN5O4 | +H | 442.1883 | 15.58 | 1.34 | 0.003 | 1/1 |
| 32 | C28H36N4O2S | +H | 493.2632 | 18.09 | 1.08 | 0.082 | 1/1 |
| 33 | C13H17NO | +H | 204.1382 | 18.34 | 1.13 | 0.084 | 1/1 |
| 34 | C28H36ClN5O3S | +H | 558.2299 | 22.03 | 0.96 | 0.490 | 1/1 |
| 35 | C24H32O7 | +H | 433.2223 | 22.78 | 1.09 | 0.062 | 1/1 |
| 36 | C15H10O2 | +H | 223.0755 | 21.21 | 0.99 | 0.889 | 1/1 |
| 37 | C15H12O2 | +H | 225.091 | 19.11 | 1.28 | 0.021 | 1/1 |
| 38 | C20H31NO2 | +H | 318.243 | 17.74 | 0.98 | 0.663 | 1/1 |
| 39 | C13H16N4O6 | +H | 325.1152 | 21.77 | 1.36 | 0.014 | 2/1 |
| 40 | C26H29ClN2O4S | +H | 501.161 | 22.01 | 1.04 | 0.302 | 1/1 |
| 41 | C26H26F3N7O2 | +H | 526.2176 | 23.1 | 1.08 | 0.304 | 1/1 |
| 42 | C25H25ClN6O4S | +H | 541.1421 | 21.88 | 1.24 | 0.010 | 1/1 |
| 43 | C25H34FN3O2 | +H | 428.2709 | 18.57 | 1.09 | 0.335 | 1/1 |
| 44 | C16H11BrN2O | +H | 327.012 | 22.3 | 1.16 | 0.073 | 1/1 |
| 45 | C30H27N5O | +H | 474.2288 | 21.77 | 1.31 | 0.022 | 1/1 |
| 46 | C14H16ClN5O2S | +H | 354.0785 | 17.9 | 0.68 | 0.260 | 1/1 |
| 47 | C28H23N7O2 | +H | 490.1985 | 16.95 | 1.21 | 0.001 | 1/1 |
| 48 | C30H26F2N4O5 | +H | 561.1949 | 24.44 | 0.87 | 0.069 | 1/1 |
| 49 | C19H17ClN2O5 | +H | 389.0899 | 17.63 | 1.07 | 0.255 | 1/1 |
| 50 | C42H45ClN6O5S2 | +H | 813.2653 | 19.72 | 0.99 | 0.946 | 1/1 |
| 51 | C20H20N4O3 | +H | 365.1609 | 15.51 | 1.12 | 0.136 | 1/1 |
| 52 | C27H31FN4O2 | +H | 463.2503 | 19.33 | 0.96 | 0.517 | 1/1 |
| 53 | C31H33ClN2O3 | +H | 517.2253 | 24.41 | 1.11 | 0.002 | 1/1 |
| 54 | C12H13N3O3S | +H | 280.0749 | 22.28 | 1.37 | 0.019 | 1/1 |
| 55 | C22H26FN3O2 | +H | 384.208 | 21.21 | 0.85 | 0.464 | 1/1 |
| 56 | C17H18Br2N2O | +H | 426.9834 | 18.55 | 1.15 | 0.032 | 1/1 |
| 57 | C17H18F3N3O3 | +H | 370.137 | 18.33 | 1.26 | 0.092 | 1/1 |
| 58 | C28H24FN3O5 | +H | 502.1774 | 16.18 | 1.07 | 0.061 | 1/1 |
| 59 | C18H22N2S | +H | 299.1578 | 16.78 | 1.16 | 0.060 | 1/1 |
| 60 | C21H25ClO6 | +NH4 | 426.1676 | 17.11 | 2.04 | 0.059 | 2/1 |
| 61 | C26H29NO4S2 | +H | 484.1613 | 15.53 | 1.08 | 0.439 | 1/1 |
| 62 | C18H11BrN2O2 | +H | 367.0074 | 24.97 | 1.27 | 0.001 | 1/1 |
| 63 | C19H12ClN3O2 | +H | 350.069 | 20.42 | 1.28 | 0.023 | 1/1 |
| 64 | C17H13Cl2NO4 | +H | 366.0291 | 17.97 | 1.06 | 0.624 | 1/1 |
| 65 | C25H27NO2 | +H | 374.2117 | 19.41 | 1.03 | 0.533 | 1/1 |
| 66 | C18H17NO4 | +H | 312.1232 | 23.7 | 0.97 | 0.332 | 1/1 |
| 67 | C15H11Cl2NO3 | +H | 324.0188 | 17.9 | 0.77 | 0.230 | 1/1 |
| 68 | C11H6F3NO2S | +H | 274.0144 | 24.92 | 1.22 | 0.187 | 1/1 |
| 69 | C21H24N4O2 | +H | 365.1973 | 21.05 | 1.01 | 0.633 | 1/1 |
| 70 | C18H23F2N5O4S2 | +H | 476.1233 | 17.71 | 1.24 | 0.001 | 1/1 |
| 71 | C24H23N3O5 | +H | 434.171 | 18.49 | 1.42 | 0.015 | 1/1 |
| 72 | C25H22F3N5O4S | +H | 546.142 | 20.18 | 1.18 | 0.022 | 1/1 |
| 73 | C28H37N3O3S3 | +H | 560.2075 | 23.83 | 1.07 | 0.670 | 1/1 |
| 74 | C35H28F3N5O2 | +H | 608.2268 | 24.99 | 1.19 | 0.010 | 1/1 |
| 75 | C18H18N2O3 | +H | 311.139 | 21.62 | 1.24 | 0.002 | 1/1 |
| 76 | C22H30FN3O7 | +H | 468.2137 | 16.94 | 1.04 | 0.205 | 1/1 |
| 77 | C20H19N3O | +H | 318.1601 | 16.74 | 0.93 | 0.003 | 1/1 |
| 78 | C16H14N2O3 | +H | 283.1076 | 21.32 | 1.12 | 0.167 | 1/1 |
| 79 | C20H28O6 | +H | 365.1963 | 21.05 | 1.01 | 0.644 | 1/1 |
| 80 | C14H10F3N5O2 | +H | 338.0857 | 15.86 | 1.37 | 0.020 | 1/1 |
| 81 | C16H16N2OS | +H | 285.1058 | 18.04 | 0.71 | 0.063 | 1/1 |
| 82 | C17H11F6N7O | +H | 444.0999 | 24.62 | 1.09 | 0.130 | 1/1 |
| 83 | C25H34N4O2S | +H | 455.2475 | 21.35 | 1.27 | 0.039 | 1/1 |
| 84 | C22H29N7O5 | +H | 472.2292 | 16.48 | 0.55 | 0.004 | 1/2 |
| 85 | C30H27BrN4O2 | +H | 555.1387 | 15.84 | 1.09 | 0.298 | 1/1 |
| 86 | C32H36N6O4 | +H | 569.2876 | 19.37 | 1.06 | 0.429 | 1/1 |
| 87 | C12H21N3O2 | +H | 240.1703 | 15.11 | 0.97 | 0.632 | 1/1 |
| 88 | C25H27N5O5 | +H | 478.2084 | 24.39 | 1.01 | 0.924 | 1/1 |
| 89 | C15H12N2O | +H | 237.1024 | 22.42 | 1.14 | 0.026 | 1/1 |
| 90 | C19H17NO3 | +H | 308.1283 | 16.78 | 1.10 | 0.107 | 1/1 |
| 91 | C17H12BrFN2O3 | +H | 391.0085 | 21.25 | 1.13 | 0.056 | 1/1 |
| 92 | C22H30N4O6 | +H | 447.2234 | 21.62 | 1.13 | 0.014 | 1/1 |
| 93 | C21H19FN4O | +H | 363.1613 | 22.41 | 1.07 | 0.277 | 1/1 |
| 94 | C23H17FN6O2 | +H | 429.147 | 17.85 | 0.85 | 0.133 | 1/1 |
| 95 | C20H15NO3 | +H | 318.1124 | 21.95 | 1.92 | 0.050 | 2/1 |
| 96 | C20H22N2O5 | +H | 371.1605 | 18.85 | 0.55 | 0.000 | 1/2 |
| 97 | C32H38ClN3O2 | +H | 532.2721 | 19.98 | 0.78 | 0.003 | 1/1 |
| 98 | C24H27N5O3 | +H | 434.2186 | 15.54 | 1.16 | 0.060 | 1/1 |
| 99 | C16H15NO7S | +H | 366.064 | 16.61 | 1.39 | 0.007 | 1/1 |
| 100 | C16H14N2O4 | +H | 299.1028 | 23.64 | 1.15 | 0.148 | 1/1 |
| 101 | C24H18F3N3O4 | +H | 470.1322 | 15.91 | 1.10 | 0.153 | 1/1 |
| 102 | C17H14ClF2N3O3S | +H | 414.0483 | 23.71 | 1.17 | 0.049 | 1/1 |
| 103 | C19H18F3N3O6 | +H | 442.1217 | 21.46 | 1.13 | 0.047 | 1/1 |
| 104 | C16H13F3N4O | +H | 335.1116 | 20.78 | 1.09 | 0.113 | 1/1 |
| 105 | C26H27N5O2 | +H | 442.2224 | 17.92 | 18.52 | 0.000 | 16/1 |
| 106 | C17H18BrN3O4S | +H | 440.027 | 21.35 | 1.20 | 0.009 | 1/1 |
| 107 | C19H17N3O2S2 | +H | 384.0835 | 24.82 | 1.37 | 0.004 | 1/1 |
| 108 | C19H18ClN5OS | +H | 400.0994 | 15.61 | 1.00 | 0.926 | 1/1 |
| 109 | C12H9ClN2O4 | +H | 281.0324 | 18.81 | 1.19 | 0.030 | 1/1 |
| 110 | C18H16N4O3S | +H | 369.1018 | 22.93 | 1.21 | 0.061 | 1/1 |
| 111 | C19H23N5O3 | +H | 370.1872 | 21.69 | 1.15 | 0.036 | 1/1 |
| 112 | C23H25N3O3S | +H | 424.169 | 16.79 | 1.23 | 0.007 | 1/1 |
| 113 | C23H24FN9O | +H | 462.2162 | 22.67 | 1.18 | 0.069 | 1/1 |
| 114 | C29H23N5O3S | +H | 522.1596 | 23.71 | 1.22 | 0.185 | 1/1 |
| 115 | C26H43N5O7 | +H | 538.3236 | 16.99 | 1.12 | 0.165 | 1/1 |
| 116 | C28H34N2O | +H | 415.2745 | 18.99 | 1.13 | 0.133 | 1/1 |
| 117 | C25H17F2N5O3S | +H | 506.1092 | 16.83 | 1.18 | 0.074 | 1/1 |
| 118 | C29H31F3N2O3 | +H | 513.2363 | 21.84 | 1.08 | 0.387 | 1/1 |
| 119 | C24H20F3N5O | +H | 452.1692 | 17.31 | 0.93 | 0.214 | 1/1 |
| 120 | C9H7ClFN5O2 | +H | 272.0344 | 15.48 | 1.21 | 0.063 | 1/1 |
| 121 | C30H28FN7O3 | +H | 554.2312 | 24.25 | 1.31 | 0.022 | 1/1 |
| 122 | C21H18FN5O | +H | 376.1569 | 22.76 | 1.21 | 0.000 | 1/1 |
| 123 | C29H27F3N6O | +H | 533.2272 | 18.3 | 1.27 | 0.002 | 1/1 |
| 124 | C19H14N2O4 | +Na | 357.0843 | 15.04 | 1.73 | 0.011 | 2/1 |
| 125 | C29H24F3N5O | +NH4 | 533.2272 | 18.3 | 1.27 | 0.002 | 1/1 |
| 126 | C15H22N2O | +H | 247.1804 | 24.68 | 1.41 | 0.069 | 1/1 |
| 127 | C19H18N2O4S | +H | 371.1059 | 23.76 | 1.21 | 0.001 | 1/1 |
| 128 | C24H26FNO4 | +H | 412.1958 | 18.18 | 1.12 | 0.221 | 1/1 |
| 129 | C15H24N2O | +H | 249.196 | 18.99 | 1.50 | 0.034 | 1/1 |
| 130 | C22H22F3N3O3 | +H | 434.1698 | 18.48 | 1.39 | 0.021 | 1/1 |
| 131 | C12H17N3O3S | +H | 284.1099 | 21.32 | 1.16 | 0.202 | 1/1 |
| 132 | C26H22FN7O3 | +H | 500.1895 | 24.39 | 1.12 | 0.007 | 1/1 |
| 133 | C25H27NO2 | +H | 374.2115 | 18.53 | 1.12 | 0.037 | 1/1 |
| 134 | C18H17NO4 | +H | 312.123 | 21.26 | 1.12 | 0.011 | 1/1 |
| 135 | C18H16FN3O | +H | 310.1342 | 16.77 | 1.14 | 0.161 | 1/1 |
| 136 | C9H9N3O2 | +H | 192.075 | 20.56 | 1.19 | 0.089 | 1/1 |
| 137 | C21H20N4O3 | +H | 377.1599 | 22.76 | 1.20 | 0.003 | 1/1 |
| 138 | C8H10FN3O3S | +H | 248.0487 | 22.93 | 1.17 | 0.566 | 1/1 |
| 139 | C29H31N7O | +H | 494.2657 | 18.09 | 1.04 | 0.445 | 1/1 |
| 140 | C19H16N8O | +H | 373.1569 | 18.85 | 0.55 | 0.000 | 1/2 |
| 141 | C19H17N3O3 | +H | 336.1341 | 24.39 | 1.12 | 0.097 | 1/1 |
| 142 | C16H13FN2O2 | +H | 285.1049 | 18.04 | 0.72 | 0.061 | 1/1 |
| 143 | C17H15FN2O3 | +H | 315.115 | 22.32 | 1.03 | 0.784 | 1/1 |
| 144 | C17H15FN2O3 | +H | 315.1139 | 24.44 | 1.02 | 0.787 | 1/1 |
| 145 | C20H31N3O2S2 | +H | 410.1984 | 18.19 | 1.19 | 0.232 | 1/1 |
| 146 | C24H27N5O3 | +H | 434.2243 | 22.78 | 1.09 | 0.033 | 1/1 |
| 147 | C19H23N9O | +H | 394.2107 | 16.79 | 1.15 | 0.141 | 1/1 |
| 148 | C14H12N4OS | +H | 285.0803 | 23.71 | 1.03 | 0.713 | 1/1 |
| 149 | C18H25N5S2 | +H | 376.1572 | 22.76 | 1.26 | 0.001 | 1/1 |
| 150 | C20H12N2O3S | +Na | 383.0469 | 19.13 | 1.16 | 0.023 | 1/1 |
| 151 | C17H12N2O2 | +Na | 299.0792 | 23.27 | 1.37 | 0.000 | 1/1 |
| 152 | C19H20N4O2 | +Na | 359.1428 | 17.22 | 0.97 | 0.692 | 1/1 |

1. GC-MS standard mixture dataset

Table S2. Concentrations of 33 amino acids in 7 mixtures: (concentration unit: μM)

| 1 | Alanine | 20 | 40 | 200 | 400 | 800 | 1200 | 1600 |
| --- | --- | --- | --- | --- | --- | --- | --- | --- |
| 2 | Sarcosine | 1 | 2 | 10 | 20 | 40 | 60 | 80 |
| 3 | Valine | 10 | 20 | 100 | 200 | 400 | 600 | 800 |
| 4 | Leucine | 5 | 10 | 50 | 100 | 200 | 300 | 400 |
| 5 | Isoleucine | 5 | 10 | 50 | 100 | 200 | 300 | 400 |
| 6 | Proline | 10 | 20 | 100 | 200 | 400 | 600 | 800 |
| 7 | Glycine | 25 | 50 | 250 | 500 | 1000 | 1500 | 2000 |
| 8 | Serine | 5 | 10 | 50 | 100 | 200 | 300 | 400 |
| 9 | threonine | 5 | 10 | 50 | 100 | 200 | 300 | 400 |
| 10 | asparagine | 5 | 10 | 50 | 100 | 200 | 300 | 400 |
| 11 | Aspartic acid | 5 | 10 | 50 | 100 | 200 | 300 | 400 |
| 12 | L-Methionine | 5 | 10 | 50 | 100 | 200 | 300 | 400 |
| 13 | cis-4-Hydroxy-L-proline | 1 | 2 | 10 | 20 | 40 | 60 | 80 |
| 14 | Creatinine | 10 | 20 | 100 | 200 | 400 | 600 | 800 |
| 15 | Phenylethylamine | 0.1 | 0.2 | 1 | 2 | 4 | 6 | 8 |
| 16 | Glutamic acid | 10 | 20 | 100 | 200 | 400 | 600 | 800 |
| 17 | phenylalanine | 5 | 10 | 50 | 100 | 200 | 300 | 400 |
| 18 | Acetylornithine | 0.5 | 1 | 5 | 10 | 20 | 30 | 40 |
| 19 | 2-Aminoadipic acid | 1 | 2 | 10 | 20 | 40 | 60 | 80 |
| 20 | Putrescine | 0.1 | 0.2 | 1 | 2 | 4 | 6 | 8 |
| 21 | Glutamine | 20 | 40 | 200 | 400 | 800 | 1200 | 1600 |
| 22 | Ornithine | 5 | 10 | 50 | 100 | 200 | 300 | 400 |
| 23 | Citrulline | 5 | 10 | 50 | 100 | 200 | 300 | 400 |
| 24 | Histamine | 1 | 2 | 10 | 20 | 400 | 600 | 800 |
| 25 | Histidine | 5 | 10 | 50 | 100 | 200 | 300 | 400 |
| 26 | Lysine | 10 | 20 | 100 | 200 | 400 | 600 | 800 |
| 27 | Tyrosine | 5 | 10 | 50 | 100 | 200 | 300 | 400 |
| 28 | dopamine | 1 | 2 | 10 | 20 | 40 | 60 | 80 |
| 29 | dopa | 0.5 | 1 | 5 | 10 | 20 | 30 | 40 |
| 30 | kynurenine | 1 | 2 | 10 | 20 | 40 | 60 | 80 |
| 31 | Tryptophan | 5 | 10 | 50 | 100 | 200 | 300 | 400 |
| 32 | Serotonin | 0.1 | 0.2 | 1 | 2 | 4 | 6 | 8 |
| 33 | Spermine | 0.25 | 0.5 | 2.5 | 5 | 10 | 15 | 20 |

All the mixtures were analyzed using a GC/TOF-MS (Leco, U.S.A.) platform in our lab.

1. LC-MS and GC-MS real-world datasets

Two real world data sets from our previous animal study were used to validate the performance of peak table statistical analysis and interpretation[2]. Metabolic profilings were acquired using UPLC/QTOF-MS (Waters, U.S.A.; positive and negative modes) and GC/TOF-MS (Leco, U.S.A.) platforms and preprocessed by Progenesis QI (Waters, U.S.A.) and ChromaTOF (Leco, U.S.A.) respectively. Gut microbiota 16S rRNA (V3) of all the intestinal content samples was amplified and measured using the IlluminaMiseq platform.

All animal handling and experiments were performed strictly in accordance with the recommendations of the Guide for the Care and Use of Laboratory Animals from the National Institutes of Health.

Results

1. Parameters of other tools

Comparisons of the peak picking performance of IP4M and the other tools are based on the standard mixtures data sets from GC/MS and LC/MS platforms respectively.

There are 6 tools involved in LC/MS data preprocessing. Their default parameters are shown in below tables.

Table S3. Parameters and the default values of Metaboseek.

| Peak detection | | Default value | | Peak filling | | | Default value | |  |
| --- | --- | --- | --- | --- | --- | --- | --- | --- | --- |
| Ppm | | 10 | | expandMz | | | 0.005 | |  |
| Peakwidth | | 3 20 | | expandRt | | | 5 | |  |
| snthresh | | 3 | | ppm | | | 3 | |  |
| prefilter | | 3 100 | | ppm_m | | | 5 | |  |
| fitgauss | | FALSE | | rtw | | | 5 | |  |
| integrate | | 1 | | rtrange | | | TRUE | |  |
| firstBaselineCheck | | TRUE | |  | | |  | |  |
| noise | | 0 | |  | | |  | |  |
| mzCenterFun | | wMean | |  | | |  | |  |
| mzdiff | | -0.005 | |  | | |  | |  |
| workers | | 1 | |  | | |  | |  |
| Feature grouping | Default  value | | CAMERA | | Default value | RT correction | | Default  value | |
| minfrac | 0.2 | | polarity | | Positive | method | | obiwarp | |
| bw | 10 | | ppm | | 10 | profStep | | 0.1 | |
| mzwid | 0.05 | | mzabs | | 0.005 | response | | 1 | |
| max | 500 | | sigma | | 3 | distFunc | | cor_opt | |
| minsamp | 1 | | perfwhm | | 0.5 | minFraction | | 0.9 | |
| usegroups | FALSE | | cor_wic_th | | 0 | extraPeaks | | 1 | |
|  |  | | pval | | 0.05 | smooth | | Loess | |
|  |  | | maxcharge | | 3 | span | | 0.2 | |
|  |  | | maxiso | | 4 | family | | gaussian | |
|  |  | | minfrac | | 0 |  | |  | |
|  |  | | filter | | FALSE |  | |  | |

Table S4. Parameters and the default values of Galaxy-M.

| ALL | Default value |
| --- | --- |
| step | 0.02 |
| snthresh | 3 |
| mzdiff | 0.05 |
| bw | 10 |
| mzwid | 0.05 |
| cor_eic_th | 0 |

Table S5. Parameters and the default values of MetaboanalystR.

| peak picking | Default value | CAMERA | Default value |
| --- | --- | --- | --- |
| ppm | 10 | polarity | “positive” |
| min_pkw | 10 | perf.whm | 0.6 |
| max_pkw | 60 | mz.abs.iso | 0.005 |
| sn_thresh | 6 | max.charge | 2 |
| mzdiff | 0.05 | max.iso | 2 |
| bw | 10 | corr.eic.th | 0 |
| min_frac | 0.5 | mz.abs.add | 0.001 |
| min_sample_num | 1 |  |  |
| max_feats | 100 |  |  |
| bin_size | 1 |  |  |
| rt_filt | FALSE |  |  |
| rt_min | 200 |  |  |
| rt_max | 1000 |  |  |

Table S6. Parameters and the default values of W4M.

| findChromPeaks | Default  value | | groupChromPeaks | | Default  value | | adjustRtime | Default  value |
| --- | --- | --- | --- | --- | --- | --- | --- | --- |
| filterAcquisitionNum | \ | | grouping method | | PeakDensity | | method | PeakGroups |
| filterRt | \ | | bw | | 10 | | minFraction | 0.9 |
| filterMz | \ | | minFraction | | 0.5 | | extraPeaks | 1 |
| extraction method | MatchedFilter | | minSamples | | 1 | | smooth method | Loess |
| fwhm | 30 | | binSize | | 0.25 | | span | 0.2 |
| binSize | 0.1 | | maxFeatures | | 50 | | family | gaussian |
| impute | None | | Get the Peak List | | No | |  |  |
| sigma | \ | |  | |  | |  |  |
| max | 5 | |  | |  | |  |  |
| snthresh | 10 | |  | |  | |  |  |
| steps | 2 | |  | |  | |  |  |
| mzdiff | 0.05 | |  | |  | |  |  |
| fillChromPeaks | | Default value | | CAMERA | | Default value | |  |
| expandMz | | 0 | | sigma | | 6 | |  |
| expandRt | | 0 | | perfwhm | | 0.6 | |  |
| ppm | | 0 | | ppm | | 10 | |  |
| Convert retention time (seconds) into minutes | | No | | mzabs | | 0.015 | |  |
| Number of decimal places for mass values reported in ions' identifiers | | 4 | | maxcharge | | 3 | |  |
| Number of decimal places for retention time values reported in ions' identifiers | | 0 | | maxiso | | 4 | |  |
| Reported intensity values | | into | | minfrac | | 0.5 | |  |
| Replace the remain NA by 0 in the dataMatrix | | yes | | cor_eic_th | | 0 | |  |
|  | |  | | graphMethod | | Hcs | |  |
|  | |  | | pval | | 0.05 | |  |
|  | |  | | calcCiS | | Yes | |  |
|  | |  | | calcIso | | No | |  |
|  | |  | | calcCaS | | No | |  |
|  | |  | | polarity | | negative | |  |
|  | |  | | max_peaks | | 100 | |  |
|  | |  | | multiplier | | FALSE | |  |
|  | |  | | Convert retention time (seconds) into minutes | | No | |  |
|  | |  | | Number of decimal places for mass values reported in ions' identifiers. | | 4 | |  |
|  | |  | | Number of decimal places for retention time values reported in ions' identifiers | | 0 | |  |
|  | |  | | Use a personal ruleset file | | FALSE | |  |
|  | |  | | mode | | All functions | |  |

Table S7. Parameters and the default values of XCMS online.

| Feature detection | Default value | Retention time correction | Default value |
| --- | --- | --- | --- |
| FWHM | 30 | bw | 10 |
| Step | 0.1 | mzwid | 0.015 |
| mzdiff | 0.05 | minfrac | 1 |
| S/N ratio cutoff | 3 | minsamp | 1 |
| Max # chrom.peaks | 10 |  |  |
| Alignment | Default value |  |  |
| bw | 10 |  |  |
| minfrac | 5 |  |  |
| mzwid | 0.05 |  |  |
| minsamp | 1 |  |  |
| max | 100 |  |  |

Table S8. Parameters and the default values of MZmine2.

| Feature detection  Chromatogram deconvolution | Default value | CAMERA | Default value |
| --- | --- | --- | --- |
| Algorithm | Baseline cut-off | FWHM sigma | 0.2 |
| m/z center calculation | MEDIAN | FWHM percentage | 1% |
| Min peak height | \ | Isotopes max charge | 3 |
| Peak duration range(min) | 0-10 | Isotopes max per cluster | 4 |
| Baseline level | \ | Isotopes mass tolerance | \ |
|  |  | Correlation threshold | 0.9 |
|  |  | Correlation p-value | 0.05 |
|  |  | Ionization Polarity | positive |
|  |  | Do not split isotopes | yes |
|  |  | Group peaks by | Isotope ID |
|  |  | Include singletons | no |

Table S9. Parameters and the default values of MS-DAIL for LC-MS data.

| Data collection | Default parameters | Peak detection parameters | Default parameters |
| --- | --- | --- | --- |
| MS1 tolerance | 0.01 Da | Minimun peak heights | 1000 amplitude |
| Retention time begin | 0 min | Mass slice width | 0.1 Da |
| Retention time end | 100 min | Smoothing method | Linear weighted moving average |
| MS1 mass range begin | 0 Da | Smoothing level | 3 scan |
| MS1 mass range end | 2000 Da | Minimum peak width | 5 scan |
| Maximum charged number | 2 |  |  |
| Consider Cl and Br elements | F |  |  |
| Number of threads | 1 |  |  |
| Execute retention time corrections | F |  |  |
| Identification | Default parameters | Alignment | Default parameters |
| Retention time tolerance | 100 min | Retention time tolerance | 0.05 min |
| Accurate mass tolerance (MS1) | 0.01 Da | MS1 tolerance | 0.015 Da |
| Accurate mass tolerance (MS2) | 0.05 Da | Retention time factor | 0.5 |
| Identification score cut off | 80% | MS1 factor | 0.5 |
| Use retention time for scoring | F | Peak count filter | 0% |
| Use retention time for filtering | F | N% detected in at least one group | 0% |
| Retention time tolerance | 0.3 min | Remove features based on blank information | F |
| Accurate mass tolerance | 0.005 Da | Sample max/blank average | 5 fold change |
| Identification score cut off | 70% | Keep reference matched metabolite feature | T |
| Relative abundance cut off | 0% | Keep removable features and assign the tag | T |
| Only report the top hit | F | Gap filling by compulsion | T |

Table S10. Parameters and the default values of MS-DAIL for GC-MS data.

| Data collection |  | | Peak detection parameters |  |
| --- | --- | --- | --- | --- |
| Mass range begin | 0 Da | | Minimum peak height | 1000 amplitude |
| Mass range end | 1000 Da | | Accurate MS | F |
| Retention time begin | 0 min | | Mass slice width | 0.1 Da |
| Retention time end | 100 min | | Mass accuracy for centroiding | 0.025 Da |
| Number of threads | 1 | | Smoothing methods | Linear weighted moving average |
|  |  | | Smoothing level | 3 scan |
|  |  | | Average peak width | 20 scan |
| Identification | |  | Alignment |  |
| RI or RT | | RT | RI or RT | RT |
| Index type | | Alkanes | Retention time tolerance | 0.075 min |
| Retention index tolerance | | 20 | EI similarity tolerance | 70% |
| Retention time tolerance | | 0.5 min | Retention time factor | 0.5 |
| m/z tolerance | | 0.5 Da | EI similarity factor | 0.5 |
| EI similarity cut off | | 70% | Identification after alignment | F |
| Identification score cut off | | 70% | Gap filling by compulsion | T |
| Use retention information for scoring | | T | Choose base peak’s m/z for rep.quant mass | F |
| Use retention information for filtering | | F |  |  |
| Use quant masses defined in MSP format file | | F |  |  |
| Only report the top hit | | T |  |  |
| Filtering | |  |  |  |
| Peak count filter | | 0 % |  |  |
| Remove feature based on blank information | | F |  |  |
| Sample max/blank average | | 5 fold change |  |  |
| Keep reference matched metabolite features | | T |  |  |
| Keep removable features and assign the tag | | T |  |  |
| Peak count filter | | 0 % |  |  |
| Remove feature based on blank information | | F |  |  |

1. Pathway and enrichment analysis

Table S11. 67 species for pathway analysis.

|  | organism | species | phylogeny |
| --- | --- | --- | --- |
| 1 | ptr | Pan troglodytes (chimpanzee) | Eukaryotes;Animals;Vertebrates;Mammals |
| 2 | pps | Pan paniscus (bonobo) | Eukaryotes;Animals;Vertebrates;Mammals |
| 3 | ggo | Gorilla gorilla gorilla (western lowland gorilla) | Eukaryotes;Animals;Vertebrates;Mammals |
| 4 | pon | Pongo abelii (Sumatran orangutan) | Eukaryotes;Animals;Vertebrates;Mammals |
| 5 | nle | Nomascus leucogenys (northern white-cheeked gibbon) | Eukaryotes;Animals;Vertebrates;Mammals |
| 6 | mcc | Macaca mulatta (rhesus monkey) | Eukaryotes;Animals;Vertebrates;Mammals |
| 7 | mcf | Macaca fascicularis (crab-eating macaque) | Eukaryotes;Animals;Vertebrates;Mammals |
| 8 | cge | Cricetulus griseus (Chinese hamster) | Eukaryotes;Animals;Vertebrates;Mammals |
| 9 | hgl | Heterocephalus glaber (naked mole rat) | Eukaryotes;Animals;Vertebrates;Mammals |
| 10 | ocu | Oryctolagus cuniculus (rabbit) | Eukaryotes;Animals;Vertebrates;Mammals |
| 11 | cfa | Canis familiaris (dog) | Eukaryotes;Animals;Vertebrates;Mammals |
| 12 | bbub | Bubalus bubalis (water buffalo) | Eukaryotes;Animals;Vertebrates;Mammals |
| 13 | chx | Capra hircus (goat) | Eukaryotes;Animals;Vertebrates;Mammals |
| 14 | oas | Ovis aries (sheep) | Eukaryotes;Animals;Vertebrates;Mammals |
| 15 | ssc | Sus scrofa (pig) | Eukaryotes;Animals;Vertebrates;Mammals |
| 16 | ecb | Equus caballus (horse) | Eukaryotes;Animals;Vertebrates;Mammals |
| 17 | myb | Myotis brandtii (Brandt's bat) | Eukaryotes;Animals;Vertebrates;Mammals |
| 18 | pcw | Phascolarctos cinereus (koala) | Eukaryotes;Animals;Vertebrates;Mammals |
| 19 | fab | Ficedula albicollis (collared flycatcher) | Eukaryotes;Animals;Vertebrates;Birds |
| 20 | cpic | Chrysemys picta (western painted turtle) | Eukaryotes;Animals;Vertebrates;Reptiles |
| 21 | gja | Gekko japonicus | Eukaryotes;Animals;Vertebrates;Reptiles |
| 22 | ipu | Ictalurus punctatus (channel catfish) | Eukaryotes;Animals;Vertebrates;Fishes |
| 23 | tru | Takifugu rubripes (torafugu) | Eukaryotes;Animals;Vertebrates;Fishes |
| 24 | der | Drosophila erecta | Eukaryotes;Animals;Arthropods;Insects |
| 25 | ame | Apis mellifera (honey bee) | Eukaryotes;Animals;Arthropods;Insects |
| 26 | bim | Bombus impatiens (common eastern bumble bee) | Eukaryotes;Animals;Arthropods;Insects |
| 27 | mpha | Monomorium pharaonis (pharaoh ant) | Eukaryotes;Animals;Arthropods;Insects |
| 28 | bmor | Bombyx mori (domestic silkworm) | Eukaryotes;Animals;Arthropods;Insects |
| 29 | dpl | Danaus plexippus (monarch butterfly) | Eukaryotes;Animals;Arthropods;Insects |
| 30 | haw | Helicoverpa armigera (cotton bollworm) | Eukaryotes;Animals;Arthropods;Insects |
| 31 | csab | Chlorocebus sabaeus (green monkey) | Eukaryotes;Animals;Vertebrates;Mammals |
| 32 | rro | Rhinopithecus roxellana (golden snub-nosed monkey) | Eukaryotes;Animals;Vertebrates;Mammals |
| 33 | rbb | Rhinopithecus bieti (black snub-nosed monkey) | Eukaryotes;Animals;Vertebrates;Mammals |
| 34 | sbq | Saimiri boliviensis boliviensis (Bolivian squirrel monkey) | Eukaryotes;Animals;Vertebrates;Mammals |
| 35 | mcal | Mus caroli (Ryukyu mouse) | Eukaryotes;Animals;Vertebrates;Mammals |
| 36 | mpah | Mus pahari (shrew mouse) | Eukaryotes;Animals;Vertebrates;Mammals |
| 37 | mun | Meriones unguiculatus (Mongolian gerbil) | Eukaryotes;Animals;Vertebrates;Mammals |
| 38 | ngi | Nannospalax galili (Upper Galilee mountains blind mole rat) | Eukaryotes;Animals;Vertebrates;Mammals |
| 39 | fca | Felis catus (domestic cat) | Eukaryotes;Animals;Vertebrates;Mammals |
| 40 | bacu | Balaenoptera acutorostrata scammoni (minke whale) | Eukaryotes;Animals;Vertebrates;Mammals |
| 41 | myd | Myotis davidii | Eukaryotes;Animals;Vertebrates;Mammals |
| 42 | amj | Alligator mississippiensis (American alligator) | Eukaryotes;Animals;Vertebrates;Reptiles |
| 43 | xla | Xenopus laevis (African clawed frog) | Eukaryotes;Animals;Vertebrates;Amphibians |
| 44 | mmu | Mus musculus (mouse) | Eukaryotes;Animals;Vertebrates;Mammals |
| 45 | rno | Rattus norvegicus (rat) | Eukaryotes;Animals;Vertebrates;Mammals |
| 46 | bta | Bos taurus (cow) | Eukaryotes;Animals;Vertebrates;Mammals |
| 47 | gga | Gallus gallus (chicken) | Eukaryotes;Animals;Vertebrates;Birds |
| 48 | dre | Danio rerio (zebrafish) | Eukaryotes;Animals;Vertebrates;Fishes |
| 49 | dme | Drosophila melanogaster (fruit fly) | Eukaryotes;Animals;Arthropods;Insects |
| 50 | cel | Caenorhabditis elegans (nematode) | Eukaryotes;Animals;Nematodes |
| 51 | ath | Arabidopsis thaliana (thale cress) | Eukaryotes;Plants;Eudicots;Mustard family |
| 52 | sce | Saccharomyces cerevisiae (budding yeast) | Eukaryotes;Fungi;Ascomycetes;Saccharomycetes |
| 53 | pfa | Plasmodium falciparum 3D7 | Eukaryotes;Protists;Alveolates;Apicomplexans |
| 54 | tbr | Trypanosoma brucei | Eukaryotes;Protists;Euglenozoa;Kinetoplasts |
| 55 | eco | Escherichia coli K-12 MG1655 | Prokaryotes;Bacteria;Gammaproteobacteria - Enterobacteria;Escherichia |
| 56 | kpn | Klebsiella pneumoniae subsp. pneumoniae MGH 78578 (serotype K52) | Prokaryotes;Bacteria;Gammaproteobacteria - Enterobacteria;Klebsiella |
| 57 | kpv | Klebsiella pneumoniae subsp. pneumoniae KPNIH29 | Prokaryotes;Bacteria;Gammaproteobacteria - Enterobacteria;Klebsiella |
| 58 | kva | Klebsiella variicola At-22 | Prokaryotes;Bacteria;Gammaproteobacteria - Enterobacteria;Klebsiella |
| 59 | ppu | Pseudomonas putida KT2440 | Prokaryotes;Bacteria;Gammaproteobacteria - Others;Pseudomonas |
| 60 | mln | Mesorhizobium loti NZP2037 | Prokaryotes;Bacteria;Alphaproteobacteria;Mesorhizobium |
| 61 | bsu | Bacillus subtilis subsp. subtilis 168 | Prokaryotes;Bacteria;Firmicutes - Bacilli;Bacillus |
| 62 | syf | Synechococcus elongatus PCC7942 | Prokaryotes;Bacteria;Cyanobacteria;Synechococcus |
| 63 | smm | Schistosoma mansoni | Eukaryotes;Animals;Flatworms |
| 64 | tma | Thermotoga maritima MSB8 | Prokaryotes;Bacteria;Thermotogae;Thermotoga |
| 65 | sau | Staphylococcus aureus subsp. aureus N315 (MRSA/VSSA) | Prokaryotes;Bacteria;Firmicutes - Bacilli;Staphylococcus |
| 66 | spym | Streptococcus pyogenes M1 476 (serotype M1) | Prokaryotes;Bacteria;Firmicutes - Bacilli;Streptococcus |
| 67 | hsa | Homo sapiens (human) | Eukaryotes;Animals;Vertebrates;Mammals |

1. Typical multivariable analysis results of some other tools

As figure S1 and S2 show, significant difference between the young group (G1) and the mature group (G2) were confirmed by both Metaboanalyst and W4M. These results are highly consistent with those of IP4M.


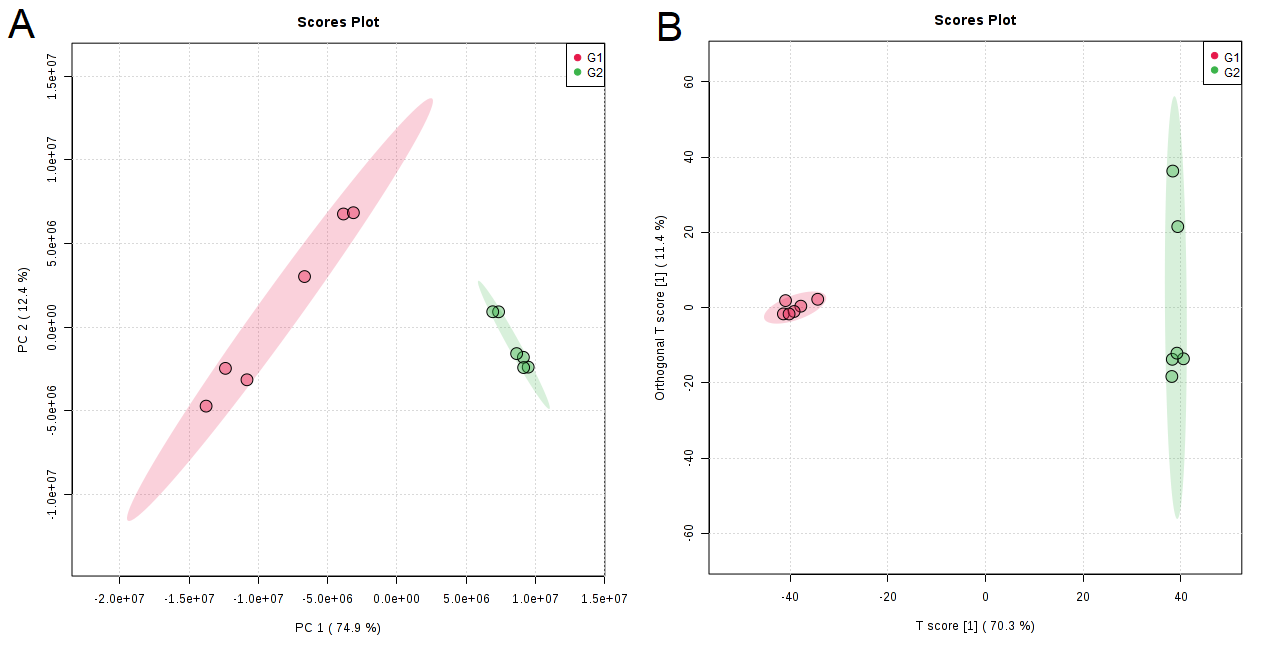


Figure S1 Multivariable analysis results of Metaboanalyst based on real world dataset. ‘G1’ means the group of one-week-old mice and ‘G2’ means the group of seven-week-old mice. (A), PCA scores plot; (B), OPLS-DA scores plot.


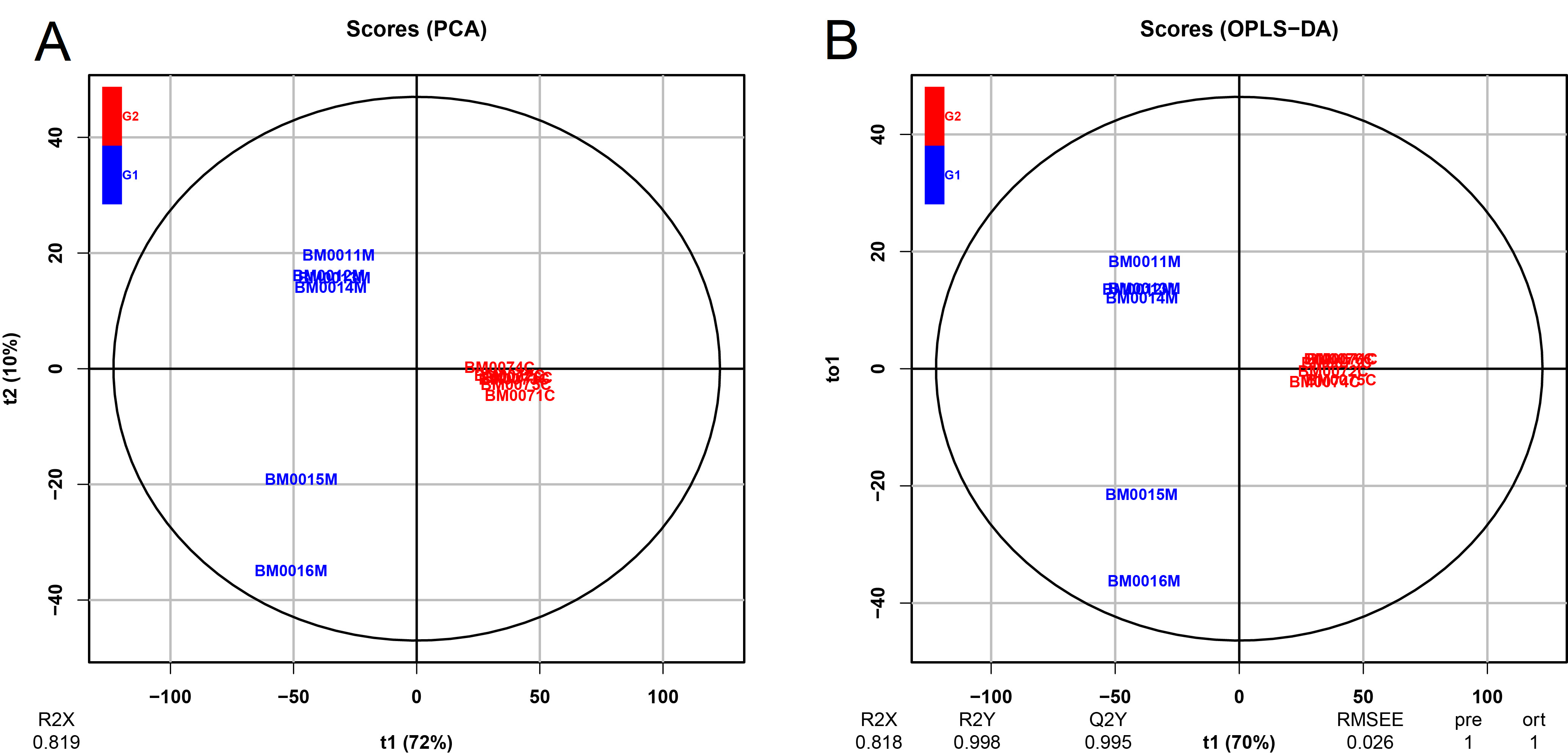


Figure S2 Multivariable analysis results of W4M based on real world dataset. ‘G1’ means the group of one-week-old mice and ‘G2’ means the group of seven-week-old mice. (A), PCA scores plot; (B), OPLS-DA scores plot.

**Reference**

1. Li Z, Lu Y, Guo Y, Cao H, Wang Q, Shui WJACA. Comprehensive evaluation of untargeted metabolomics data processing software in feature detection, quantification and discriminating marker selection.S0003267018305725.

2. Chen T, You Y, Xie G, Zheng X, Zhao A, Liu J, et al. Strategy for an Association Study of the Intestinal Microbiome and Brain Metabolome Across the Lifespan of Rats. Anal Chem. 2018;90(4):2475-83.
